# A randomized study of pomalidomide vs placebo in persons with myeloproliferative neoplasm-associated myelofibrosis and RBC-transfusion dependence

**DOI:** 10.1038/leu.2016.300

**Published:** 2016-11-18

**Authors:** A Tefferi, H K Al-Ali, G Barosi, T Devos, H Gisslinger, Q Jiang, J-J Kiladjian, R Mesa, F Passamonti, V Ribrag, G Schiller, A M Vannucchi, D Zhou, D Reiser, J Zhong, R P Gale, for the RESUME trialists

**Affiliations:** 1 Division of Hematology, Department of Internal Medicine, Mayo Clinic College of Medicine, Rochester, MN, USA; 2 Universitätsklinikum Leipzig, Leipzig, Germany; 3 Fondazione IRCCS Policlinico S Matteo, Pavia, Italy; 4 Universitaire Ziekenhuizen, Leuven, Belgium; 5 Medizinische Universität Wien, Vienna, Austria; 6 Peking University People's Hospital, Beijing, China; 7 Hôpital Saint-Louis, Paris, France; 8 Mayo Clinic, Scottsdale, AZ, USA; 9 University of Insubria, Varese, Italy; 10 Gustave Roussy, Université Paris-Saclay, Saint-Aubin, France; 11 Division of Hematology and Oncology, David Geffen School of Medicine at UCLA, Los Angeles, CA, USA; 12 Centro Ricerca e Innovazione Malattie Mieloproliferative-CRIMM, Azienda Ospedaliera Universitaria Careggi, Florence, Italy; 13 Department of Experimental and Clinical Medicine, University of Florence, Florence, Italy; 14 Peking Union Medical College Hospital, Beijing, China; 15 Celgene Corporation, Summit, NJ, USA; 16 Haematology Research Centre, Imperial College London, London, UK

## Abstract

RBC-transfusion dependence is common in persons with myeloproliferative neoplasm (MPN)-associated myelofibrosis. The objective of this study was to determine the rates of RBC-transfusion independence after therapy with pomalidomide vs placebo in persons with MPN-associated myelofibrosis and RBC-transfusion dependence. Two hundred and fifty-two subjects (intent-to-treat (ITT) population) including 229 subjects confirmed by central review (modified ITT population) were randomly assigned (2:1) to pomalidomide or placebo. Trialists and subjects were blinded to treatment allocation. Primary end point was proportion of subjects achieving RBC-transfusion independence within 6 months. One hundred and fifty-two subjects received pomalidomide and 77 placebo. Response rates were 16% (95% confidence interval (CI), 11, 23%) vs 16% (8, 26% *P*=0.87). Response in the pomalidomide cohort was associated with ⩽4 U RBC/28 days (odds ratio (OR)=3.1; 0.9, 11.1), age ⩽65 (OR=2.3; 0.9, 5.5) and type of MPN-associated myelofibrosis (OR=2.6; 0.7, 9.5). Responses in the placebo cohort were associated with ⩽4 U RBC/28 days (OR=8.6; 0.9, 82.3), white blood cell at randomization >25 × 10^9^/l (OR=4.9; 0.8, 28.9) and interval from diagnosis to randomization >2 years (OR=4.9; 1.1, 21.9). Pomalidomide was associated with increased rates of oedema and neutropenia but these adverse effects were manageable. Pomalidomide and placebo had similar RBC-transfusion-independence response rates in persons with MPN-associated RBC-transfusion dependence.

## Introduction

Myeloproliferative neoplasm (MPN)-associated myelofibrosis is a clinical entity comprised of primary myelofibrosis, post-polycythaemia myelofibrosis and post-essential thrombocythaemia myelofibrosis.^1^ Anaemia is common in persons with MPN-associated myelofibrosis with frequencies of 30–100% in several large series (reviewed in references Cervantes *et al.*^2^ and Passamonti *et al.*^3^). Anaemia and RBC-transfusion dependence are associated with poorer survival.^2, 3, 4, 5, 6^ RBC transfusions are often given for severe anaemia. Some persons with MPN-associated myelofibrosis also have decreased platelet levels.

There is no US Food and Drug Administration- or European Medicines Agency-approved therapy for anaemia with or without RBC-transfusion dependence in persons with MPN-associated myelofibrosis. Common therapies include corticosteroids, androgenic steroids, erythropoietin, thalidomide and lenalidomide.^7, 8, 9, 10, 11, 12, 13, 14, 15, 16, 17, 18^ None is proved effective in a double-blind randomized clinical trial. Ruxolitinib, a recently Food and Drug Administration-approved drug for myelofibrosis, typically initially worsens anaemia (reviewed in reference Santos and Verstovsek^19^).

Pomalidomide is an immune-modulating drug with diverse biological actions, including effects on inflammation, T-cell function, angiogenesis, proliferation and erythropoiesis.^20^ The pro-erythroid activity of pomalidomide is substantially greater than that of thalidomide or lenalidomide, and it is associated with less bone marrow suppression especially at low doses.^21, 22^

A phase 2 randomized Bayesian pick-the-winner study in persons with MPN-associated myelofibrosis and anaemia with or without RBC-transfusion dependence (defined using International Working Group for Myelofibrosis Research and Therapy (IWG-MRT) criteria) compared pomalidomide, 0.5 mg/day, combined with a 3-month course of prednisone, and pomalidomide, 2 mg/day, with and without prednisone against prednisone only. Response rates were similar but response duration was significantly longer with pomalidomide, 0.5 mg/day, and persisted after prednisone was discontinued. This cohort was declared the winner.^23^ A subsequent phase 1/2 study showed a similar response rate to pomalidomide without prednisone and no evidence of a higher response rate at higher pomalidomide doses.^24^ Moreover, subjects not responding to doses of pomalidomide >2 mg/day had a high response rate to pomalidomide, 0.5 mg/day. Another phase 2 study of pomalidomide, 0.5 mg/day, without prednisone reported a 24% response rate in subjects with anaemia with or without RBC-transfusion dependence with *JAK2*^V617F^ but no responders in subjects without *JAK2*^V617F^.^25^ There was also a correlation between anaemia response and pomalidomide-induced basophilia and a high platelet response rate. Based on these data, a dose of pomalidomide, 0.5 mg/day, without prednisone was selected for further study.

We sought to determine whether therapy of persons with MPN-associated myelofibrosis and RBC-transfusion dependence with pomalidomide, 0.5 mg/day, could increase the proportion of persons achieving RBC-transfusion independence compared with placebo in a randomized, double-blind parallel-group study. Because RBC-transfusion dependence and independence are not uniformly defined, we first conducted a RAND-Delphi consensus panel study to develop definitions.^26, 27^ A third response category of clinical benefit (a 50% decrease in RBC-transfusion intensity) was also defined by this process.

## Materials and methods

### Study design and objectives

This is a phase 3, multicentre, randomized, double-blind, parallel-group, placebo-controlled study with a 2:1 allocation to pomalidomide vs placebo. The hypothesis tested was whether data from this study are consistent with a model where therapy with pomalidomide results in a 30% RBC-transfusion-independence response rate compared with a 10% background rate with placebo (see ‘Sample size and power considerations' below).

### Subjects

Subjects with MPN-associated myelofibrosis, including primary myelofibrosis, post-polycythaemia vera myelofibrosis and post-essential thrombocythaemia myelofibrosis ⩾18 years of age, were eligible. Bone marrow samples were collected and reviewed postrandomization by a blinded central observer (James Vardiman; University of Chicago). Subjects had to be RBC-transfusion dependent defined as an average RBC-transfusion frequency ⩾2 U/28 days over ⩾84 days immediately prerandomization with no interval >42 days without ⩾1 RBC transfusion. Only RBC transfusions given for a haemoglobin concentration ⩽90 g/l were scored in determining eligibility. (For countries following Nordic guidelines, haemoglobin levels for RBC transfusions should not exceed 100 g/l haemoglobin.) RBC transfusions given because of bleeding or chemotherapy- or radiation-induced anaemia were also excluded. Haemoglobin concentration at randomization had to be ⩽130 g/l. Subjects should not have received thalidomide, lenalidomide, drugs which suppress bone marrow function or any investigational drug <1 month prerandomization. Androgenic steroids and erythropoietin were prohibited within 3 months prerandomization and hydroxyurea should not have been given within 6 weeks prerandomization. Detailed inclusion and exclusion criteria are presented in Supplementary Table 1. Subjects were enrolled at 72 sites, including 18 in North America, 41 in Europe (including Russia) and 11 in Asia. Co-investigators are listed at the end of the text before References. Data were collected at the trial sites except for RBC transfusions given at other sites. In this instance, detailed primary medical records were required for review including copies of pretransfusion haemoglobin concentrations and unit-specific records of any RBC transfusions given. Data were independently reviewed for accuracy prerandomization by a physician (RPG) and deputy (DR). Discordances were adjudicated or the data were rejected and scored as a failure. Subjects carried a diary into which entries regarding RBC transfusion had to be made by physicians at sites other than the treating centre.

### Intervention

Subjects were randomized to receive pomalidomide, 0.5 mg/day by mouth, or matching placebo daily by mouth. Investigational product (pomalidomide or placebo) was withheld when the haemoglobin concentration was >140 g/l. Subjects were evaluated for adverse events (AEs) at each visit using the NCI CTCAE (Version 4.0) to grade severity. Pomalidomide and placebo were interrupted or discontinued according to prospectively defined guidelines (Supplementary Table 2). There were no dose modifications but pomalidomide or placebo could be interrupted for up to 6 weeks for AEs. Use of drugs that might confound analyses of RBC-transfusion dependence such as bone marrow-suppressive drugs, hydroxyurea, anagrelide, busulfan, immune modulators, erythropoietin, androgenic steroids, iron-chelating drugs and granulocyte- and granulocyte/macrophage colony-stimulating factors were prohibited.

### Outcomes

The primary end point was the proportion of subjects achieving RBC-transfusion independence defined as ⩾84 consecutive days with no RBC transfusion. Secondary co-end points were time to becoming RBC-transfusion independent, duration of RBC-transfusion independence, survival, frequency and severity of AEs, health-care resource use and health-related quality-of-life measures (European quality of life-5D and Functional Assessment of Cancer Therapy-Anaemia). Exploratory end points were prestudy subject-, disease- and therapy-related variables correlated with RBC-transfusion independence and with response duration and serum pomalidomide concentrations. There were unprespecified, postunblinding analyses of the frequency of platelet response, defined by criteria of the IWG-MRT criteria^15^ and of clinical benefit defined in the RAND-Delphi consensus panel study.^26, 27^ This report focusses on RBC-transfusion independence, clinical benefit, platelet response and safety. Analyses of other end points will be reported.

### Sample size and power considerations

A sample size of 189 subjects (126 receiving pomalidomide and 63 receiving placebo) would have 90% power to detect the difference between a response rate of 30% in the pomalidomide cohort and 10% in the placebo cohort. The sample size calculation is based on two-sided *Pα*=0.05 and testing the difference of proportions using un-pooled estimate of variance. The study planned to randomize 210 subjects with 140 receiving pomalidomide and 70 receiving placebo to compensate for subject attrition. An additional 42 subjects were randomized because of commitments made by investigators to subjects already in screening and without access to study results. This sample size increase was approved by the Data Monitoring Committee (see section below) and by health authorities and ethics committees at participating centres.

### Randomization

Randomization was performed using a validated interactive voice response system at a remote site. Investigators called to obtain a blinded therapy assignment and were required to begin therapy within 3 days of randomization. All randomized subjects were analyzed whether or not they received therapy. The randomization sequence allocation was stratified for three variables: (1) age (⩽vs >65 years); (2) white blood cell < or ⩾25 × 10^9^/l; and (3) intensity of RBC-transfusion dependence prerandomization (⩽vs >4 U RBC/28 days averaged over the preceding 84 days). The first subject was randomized on 8 September 2010 and the last on 2 August 2012.

### Blinding

Subjects, investigators and all trial participants were blinded to therapy assignment. An unblinded independent Data Monitoring Committee was impaneled to monitor safety. There were no planned interim analyses for efficacy or futility.

### Statistical methods

Three populations were prospectively-defined: (1) an intent-to-treat (ITT) population including all randomized subjects (*N*=252); (2) a modified ITT population including all subjects with a confirmed diagnosis of MPN-associated myelofibrosis by the blinded central reviewer, confirmed RBC-transfusion dependence by the blinded study monitor and who received ⩾1 dose of pomalidomide or placebo (*N*=229); and (3) a safety population including all randomized subjects receiving ⩾1 dose of investigational product (*N*=250).

The primary efficacy end point was proportion of subjects achieving ⩾84-day RBC-transfusion independence defined as the absence of any RBC transfusion during any consecutive rolling 84-day interval (that is, days 1–84, days 2–85 and so on) by 169 days postrandomization or beginning at least 28 days before day 169. Subjects achieving ⩾84-day RBC-transfusion independence were scored as responders. Subjects discontinued from therapy for any reason without achieving RBC-transfusion independence are scored as non-responders. Efficacy analyses were based on the modified ITT population. Numbers and percentage of responders were tabulated by therapy-assignment cohort. Fisher's exact test was used to compare pomalidomide and placebo at a two-side significance level of *Pα*=0.05.

Analyses of secondary efficacy end points were also based on the modified ITT population. Analyses of the duration of RBC-transfusion independence included only subjects achieving RBC-transfusion independence. The time origin was the date RBC-transfusion independence starts which could be the date of randomization. Duration of the RBC-transfusion independence was the time origin to the date of the next RBC transfusion, which had to be ⩾84 days after the time origin. Duration of the RBC-transfusion independence was analyzed using the Kaplan–Meier method. Data were censored at the end of the treatment phase for subjects never receiving another RBC transfusion after the time origin by the end of treatment phase. Only subjects achieving RBC-transfusion independence were included in the analysis of time to response. Days from randomization to the date at which RBC-transfusion independence starts were summarized using descriptive statistics (*N*, mean, median, s.d., minimum and maximum). Day 1 was used in the analysis if the first RBC-transfusion independence began on the randomization date. Counts and percentages were used to summarize discrete variables and descriptive statistics were used to summarize continuous variables. Univariable analyses were used to identify variables correlated with response. A multivariate logistic regression model was then used to evaluate effects of potentially significant prognostic variables simultaneously and to identify the most important variables associated with response.

Safety analyses included data from all subjects receiving ⩾1 dose of investigational product (pomalidomide or placebo). AEs, causes of death, vital signs, clinical laboratory measurements and concomitant drugs were tabulated descriptively by therapy cohort. AEs were classified using the Medical Dictionary for Drug Regulatory Activities coding system. Severity of AEs was graded according to NCI CTCAE (Version 4.0) whenever possible. Subjects with AEs were tabulated by system organ class, preferred term and treatment regimen. Subjects with >1 occurrence of the same event were counted only once under each system organ class and preferred term. The AE summary includes numbers of AEs by NCI CTCAE grade, suspected treatment-related events, events resulting in withdrawal of pomalidomide or placebo, serious AEs and AEs of special interest including new cancers. The most severe grade of each preferred term reported for a subject was used for summaries of AEs by NCI CTCAE grade. Clinical laboratory measurements were also graded according to NCI CTCAE (Version 4.0). Cross tabulations were generated to summarize frequencies of severity changes from baseline to the most abnormal laboratory values during the study period. Analyses of all end points were carried out when all subjects completed the blinded therapy phase of the study, which ended 164 days after the last subject was randomized.

## Results

### Subjects

**Figure 1:**
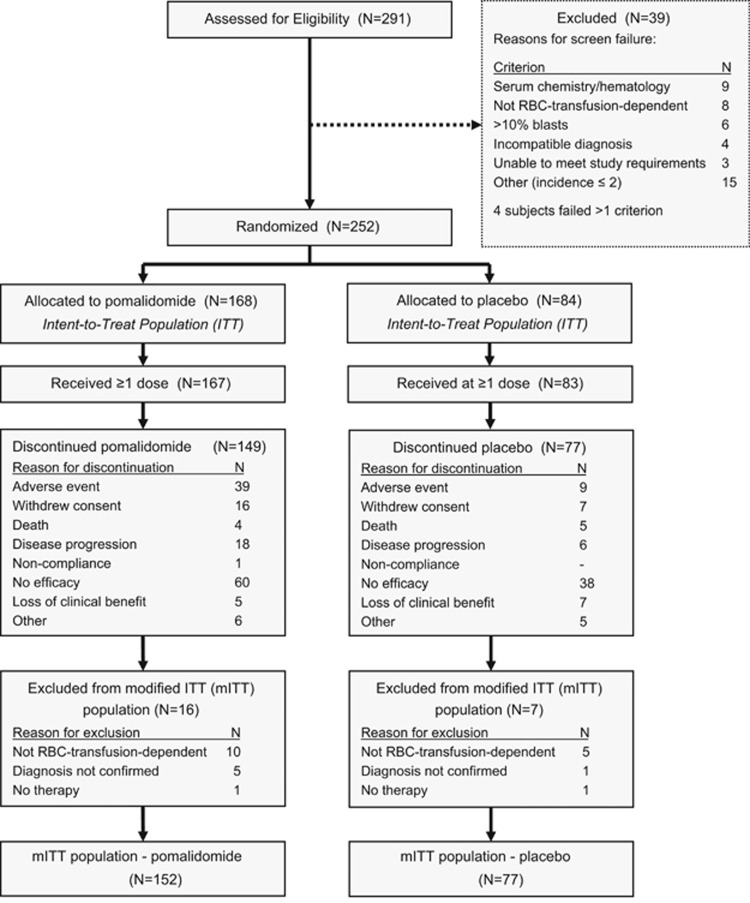
CONSORT flow diagram.

There were 252 subjects in the IIT population (Figure 1). We could not confirm the diagnosis of MPN-associated myelofibrosis at central histological review in 6 subjects nor RBC-transfusion dependence in 15. Two additional subjects received no therapy. Consequently, we focussed our analysis on the 229 subjects in the modified ITT population, including 152 in the pomalidomide cohort and 77 in the placebo cohort.

Prerandomization variables of the modified ITT population are summarized in Table 1. Median age was 70 years (range, 40–90 years). One hundred and seventy-two subjects (75%) had primary myelofibrosis, 25 (11%), post-polycythaemia vera myelofibrosis and 34 (14%), post-essential thrombocythaemia myelofibrosis. Median interval from diagnosis to randomization was 2 years (range, 0–27 years). One hundred and twenty-nine subjects (56%) received prior anaemia therapy including androgenic steroids 43 (19%), corticosteroids 67 (29%), erythropoietin 81 (35%) and other therapies 9. One hundred and ninety-three subjects (84%) had splenomegaly on physical examination and 7 had a splenectomy. Median white blood cell was 6.0 × 10^9^/l (range, 1–114 × 10^9^/l). Median RBC-transfusion units/28 days averaged over 84 days prerandomization was 3 (range, 2–13). There were no significant differences between the cohorts for any of these variables.

### RBC-transfusion independence

Response rates in the modified ITT population were similar: pomalidomide, 16% (95% confidence interval (CI), 11, 23%) vs placebo, 16% (8, 26% *P*=1.00). Median time to response for pomalidomide was 7 weeks (range, 0–20 weeks) vs 2 weeks (range, 0–15 weeks) for placebo (*P*=0.22). Median response durations were not analyzable (lower boundary of 95% CI 4.8 months) vs 5.5 months (*P*=0.44). Among 25 responders in the pomalidomide cohort, 12 had a response duration >186 days compared with 1 of the 12 responders in the placebo cohort.

Response in both cohorts was more common in subjects with low vs high U RBC/28 days prerandomization (pomalidomide OR=3.1 (0.9, 11.1); *P*=0.09; placebo (OR=8.6 ((0.9, 82.3); *P*=0.06). However, other variables associated with response to pomalidomide including age (⩽65 vs >65 years: OR=2.3 (0.9, 5.5); *P*=0.07) and type of MPN-associated myelofibrosis (primary myelofibrosis vs other: OR=2.6 (0.7, 9.5); *P*=0.14) differed from those associated with response to placebo including white blood cell (>25 vs ⩽25 × 10^9^/l; OR=4.9 (0.8, 28.9); *P*=0.08) and interval from diagnosis to randomization (>2 vs ⩽2 years; OR=4.9; (1.1, 21.9); *P*=0.04). At one centre, 4 of the 12 subjects receiving placebo responded vs 8 of the 65 at all other centres combined (*P*=0.03). This effect persisted in multivariable analyses (Supplementary Tables 3–5). However, response rates to pomalidomide and placebo remained comparable after excluding data from this centre. We also examined rates of clinical benefit (50% decrease in RBC transfusions) in an unprespecified postunblinding analysis. There was no significant difference between the cohorts.

Three pomalidomide subjects scored as responders became RBC-transfusion dependent when pomalidomide was stopped and regained RBC-transfusion independence when pomalidomide was re-started. No similar effect was seen in the placebo cohort. One additional pomalidomide subject scored as a responder achieved a haemoglobin concentration >140 g/l. Pomalidomide was stopped as specified in the protocol. The subject has a haemoglobin >140 g/l with no subsequent pomalidomide therapy for >1.5 years. No similar responses were seen in the placebo cohort. No prerandomization therapy (such as iron-chelating drugs, hydroxyurea, busulfan, folic acid) was consistently associated with response to placebo. Durations of RBC-transfusion dependence prerandomization were similar between the cohorts.

### Platelet response

Platelet response rates in subjects with baseline platelets <50 × 10^9^/l (pomalidomide, *N*=54; placebo, *N*=33) were significantly different between pomalidomide (22% (95% CI, 11, 35%)) and placebo (none (0, 12%); *P*=0.006) and was not significantly correlated with RBC-transfusion-independence response, *P*=0.69).

### Safety

Peripheral oedema, fatigue, pyrexia and diarrhoea were the most commonly reported treatment-emergent AEs. Only peripheral oedema and neutropenia were significantly more common in the pomalidomide cohort (Supplementary Table 6). The most frequently reported grade ⩾3 treatment-emergent AEs were neutropenia, thrombocytopenia, anaemia and pneumonia. There was no significant difference in incidences between the cohorts except for neutropenia, which occurred in 14% of subjects receiving pomalidomide and 6% of subjects receiving placebo (*P*=0.06).

## Discussion

There were no significant differences in the proportion of subjects with RBC-transfusion independence, time to response or response duration between subjects receiving pomalidomide vs subjects receiving placebo in the modified ITT population. Although the pomalidomide response rate was within the 95% CI estimated from our phase 2 study^23^ (albeit with brief, concurrent corticosteroids), the response rate to placebo was higher than estimated (albeit with a lower 95% confidence boundary of 8%). This high rate of seemingly spontaneous reversals of substantial RBC-transfusion dependence (median 3 U RBC/month averaged over the preceding 84 days) and brief time to response (median 2 weeks) is inconsistent with the experience of most MPN experts and literature reviews.^2, 3, 4, 5, 6^ Consequently, we searched for confounding factors such as prior exposures to diverse prerandomization and postrandomization interventions but found no consistent associations. Detailed analyses of proper coding of the investigational product and placebo was consistent with correct drug assignment for most subjects with correct investigational product drug assignment in a small cohort surveyed. Finally, the significant difference in platelet response between the cohorts favouring pomalidomide suggests correct coding and delivery of pomalidomide. Based on these data, we conclude no efficacy of pomalidomide in reversing RBC-transfusion dependence in persons with MPN-associated myelofibrosis. Interestingly, others report increased platelets in persons with MPN-associated myelofibrosis receiving pomalidomide (see accompanying article in this issue by Schlenk *et al.*).^28, 29^

One interpretation of the similar response rates to pomalidomide and placebo is that the pomalidomide responses are spontaneous reversals of RBC-transfusion dependence at a similar rate to that observed in the placebo cohort. We were therefore surprised to find several differences in variables associated with response between the cohorts. For example, age <65 years was associated with response to pomalidomide, whereas age ⩾65 years was associated with response to placebo. Another unexplained observation was the loss and recovery of response after discontinuing and restarting pomalidomide but not after discontinuing and restarting placebo. We are interrogating gene profiles in subjects in this study searching for possible associations with response to pomalidomide and to placebo.

We selected a pomalidomide dose of 0.5 mg/day based on data from phase 1/2 studies.^23, 24, 30^ In one study at the maximum tolerated dose (3 mg/day for 21 of 28 days), there were no responders but most subjects responded (cessation of RBC transfusions) when the dose was decreased to 0.5 mg/day given continuously.^24^ Based on these data, 0.5 mg/day was determined to be the effective dose. We also chose not to give prednisone because the response rate at 0.5 mg/day without prednisone in one study was comparable or higher than the response rate at the same dose using prednisone^25^ and because there was no difference in response rates at a dose of 2 mg/day with or without prednisone.^23^ In contrast to these reports, preliminary data from another phase 2 study suggest a higher response rate to 2 mg/day vs 0.5 mg/day of pomalidomide and longer response durations with concomitant prednisone.^29^ Based on these data, our conclusion of no effect of pomalidomide on RBC-transfusion dependence is restricted to the dose and schedule we tested and to its use without concomitant prednisone.

The IWG-MRT recently published a new guideline defining RBC-transfusion dependence and independence.^31^ The definition of RBC-transfusion dependence is less stringent than we used because we averaged transfusions over 84 days (which had to be ⩾6 U RBCs) and no 42-day RBC-transfusion-free interval, whereas they had no exclusionary RBC-transfusion-free 42-day interval. We also excluded subjects with a haemoglobin concentration >130 g/l at randomization, whereas there was no similar exclusion in the IWG-MRT criteria. Based on these considerations, our eligibility criteria were more stringent than those proposed by the IWG-MRT. Response criteria are similar, 84 days with no RBC transfusion. However, the IWG-MRT criteria require a haemoglobin concentration ⩾85 g/l (when is not specified, presumably at the time response is declared), whereas we had no such requirement. As a check, we re-evaluated responders in the pomalidomide and placebo cohorts and censored subjects in whom haemoglobin concentration at the time response was declared was <85 g/l. Response rates were similar to those using the RAND-Delphi criteria. We also analyzed two variables reported in a phase 2 study to correlate them with anaemia response to pomalidomide, *JAK2*^V617F^ and pomalidomide-induced basophilia.^32^ We could not confirm either association (data not shown) nor did our conclusion change when we considered response rates only in subjects with *JAK2*^V617F^.

Finally, we analyzed the validity of our conclusion regarding lack of efficacy of pomalidomide using the relevant Grading of Evidence, Assessment, Development and Evaluation (GRADE) criteria including: (1) overall risk of bias; (2) imprecision; (3) inconsistency (4) indirectness, and (5) publication bias (http://gdt.guidelinedevelopment.org/central_prod/_design/client/handbook/handbook.html). Risks of bias and imprecision are low. However, the precisely defined population of subjects with RBC-transfusion dependence we studied is not representative of all persons with MPN-associated myelofibrosis requiring therapy for anaemia (indirectness of the study population). For example, most persons with MPN-associated myelofibrosis who are RBC-transfusion dependent receive <2 U RBCs per 28 days over a ⩾3-month interval. Moreover, the rigid RBC-transfusion-independence response criteria we used do not reflect the clinical benefit physicians expect wherein a >50% reduction in RBC-transfusion intensity may be considered beneficial depending on the baseline intensity such as the 50% reduction defined as clinical benefit in our RAND-Delphi study (refer Begna *et al.*^25^; indirectness of end point). We chose our entry and response criteria based on discussions with health authorities regarding requirements for drug registration. Consequently, the strength of our conclusion of no efficacy should be downgraded according to the GRADE criteria. The GRADE sphere of publication bias is not applicable to our study as only one other randomized trial of pomalidomide in MPN-associated myelofibrosis is reported.^29^

Perhaps the most important conclusion from our study is the need for a double-blind randomized control cohort when studying interventions designed to reverse anaemia and especially RBC-transfusion dependence in persons with MPN-associated myelofibrosis and possibly other diseases where anaemia is an important issue. Results of uncontrolled studies with response rate within the 8–26% 95% CI of our placebo cohort should be viewed cautiously.

## Other RESUME clinical trialist co-investigators

The other RESUME clinical trialist co-investigators are as follows: Australia: John Catalano (Frankston Hospital, Oncology Day Unit); William Stevenson (Royal North Shore Hospital); Austria: Günther Gastl (Medizinische Universität Innsbruck); Werner Linkesch (Medizinische Universität Graz); Belgium: Jan Van Droogenbroeck (Academisch Ziekenhuis Brugge); Philippe Mineur (Grand Hôpital de Charleroi); Canada: Vikas Gupta (Princess Margaret Hospital); Andrew Turner (Cross Cancer Institute, Dept. of Medical Oncology); Thomas Nevill (Vancouver General Hospital); China: Jianyong Li (Jiangsu Province Hospital); Zhixiang Shen (Shanghai Ruijin Hospital); Ting Liu (West China Hospital, Sichuan University); France: Dominique Bordessoule (Centre Hospitalier Universitaire Limoges); Shanti Natarajan-Amé (Centre Hospitalier Universitaire de Strasbourg - Hôpital Civil); Christian Recher (Hôpital Purpan, Toulouse, Service Hématologie); Jean Loup Demory (Groupe Hospitalier de I'Institue Catholique de Lille-Hôpital Saint-Vincent de Paul (Belfort)); Germany: Richard Schlenk (Universitätsklinik Ulm); Martin Griesshammer (Johannes-Wesling-Klinikum Minden); Italy: Mario Cazzola (Fondazione IRCCS Policlinico S. Matteo); Giuseppe Saglio (Azienda Ospedaliero-Univesitaria ‘San Luigi Gonzaga'); Giorgina Specchia (Azienda Ospedaliera Policlinico di Bari); Alessandro Rambaldi (Azienda Ospedaliera Papa Giovanni XXIII); Fabrizio Pane (Azienda Ospedaliera Universitaria ‘Federico II'); Netherlands: Sonja Zweegman (VU University Medical Centre); Peter te Boekhorst (Erasmus Medisch Centrum); Reinier Raymakers (Universitair Medisch Centrum Utrecht); Russia: Kudrat Abdulkadyrov (FGU Russian Scientific Research Institute of Hematology and Transfusiology); Manana Sokolova (Hematology Scientific Center); Galina Salogub (Saint Petersburg I.P. Pavlov State Medical University); Andrey Zaritskiy (FSI ‘V.A. Almazov Federal Centre of Heart, Blood and Endocrinology of Rosmedtechnologies'); Spain: Francisco Cervantes (Hospital Clinic I Provincial de Barcelona); Juan Carlos Hernández Boluda (Hospital Clinico Universitario de Valencia); Emilio Ojeda (Hospital Puerta de Hierro Majadahonda); Sweden: Daniel Tesfa (Karolinska University Hospital Huddinge); Lars Nilsson (Skånes Universitetssjukhus i Lund); United Kingdom: Adam Mead (Churchill Hospital, Oxford); Mary McMullin (Belfast City Hospital); Mark Drummond (Beatson Oncology Centre, Glasgow); John Reilly (Royal Hallamshire Hospital, Sheffield); Claire Harrison (Guy's Hospital, London); Dragana Milojkovic (London Hammersmith Hospital); United States: Candido Rivera (Mayo Clinic, Jacksonville, FL); Emmanuel Besa (Jefferson Medical College, PA); H Joachim Deeg (Fred Hutchinson Cancer Research Center, Seattle, WA); John Mascarenhas (Mount Sinai Hospital, The Donald H Ruttenberg Cancer Treatment Center, NY); Josef Prchal (University of Utah Health Sciences Center); Ramon Tiu (The Cleveland Clinic Foundation, OH); Moshe Talpaz (University of Michigan Comprehensive Cancer Center); Jen Chin Wang (Brookdale Hospital Medical Center, NY); Raajit Rampal (Memorial Sloan-Kettering Cancer Center, NY); Damiano Rondelli (University of Illinois at Chicago); Kelly McCaul (Avera Hematology and Transplant, Sioux Falls, SD); Randall Brown (University of Florida Shands Cancer Center); Japan: Norio Komatsu (Juntendo University Hospital); Kazuma Ohyashiki (Tokyo Medical University Hospital); Kiyoshi Ando (Tokai University Hospital); Hiroshi Kawabata (Kyoto University Hospital); Katsuto Takenaka (Kyushu University Hospital); Tomoko Hata (Nagasaki University Hospital); and James Vardiman (Univ. Chicago).

## Figures and Tables

**Table 1:** Baseline variables

	*All (*N=*229)*	*Placebo (*N=*77)*	*Pomalidomide (*N=*152)*	P*-value*
Age (years; median (range))	70 (40–90)	70 (44–81)	69 (40–90)	0.47
Sex (M/F)	168	52	116	0.16
Diagnosis to randomize (years; median (range))	1.6 (0–27)	1.6 (0–14)	1.5 (0–27)	0.31
PMF	172	59	113	0.75
Post-PV-MF	25	8	17	1.00
Post-ET-MF	31	10	21	1.00
Spleen size (cm; median (range))	12 (0–30)	11 (0–27)	12 (0–30)	0.52
Haemoglobin (mg/l; median (range))	87 (40–117)	89 (54–117)	87 (40–110)	0.08
WBC (× 10^E+9/L^/l; median (range))	6.0 (0.9–114)	7.2 (1.2–114)	5.6 (0.9–67)	0.69
Platelets (× 10^E+9/L^/l; median (range))	147 (13–2108)	136 (13–2108)	157 (22–1523)	0.75
Splenectomy	7	1	6	0.52
RBC transfusions (/28 days; median (range))	3 (2–13)	3 (2–10)	3 (2–15)	0.82
RBC transfusions (>4 U/28 days)	66	25	41	0.44
No. of prior therapies (median (range))	1 (1–4)	1 (1–4)	1 (1–4)	0.82

Abbreviations: F, female; M, male; PMF, primary myelofibrosis; post-ET-MF, post-essential thrombocythemia myelofibrosis; post-PV-MF, post-polycythaemia vera myelofibrosis; RBC, red blood cell; WBC, white blood cell.
