# Supplementary material for: A randomized study of pomalidomide vs placebo in persons with myeloproliferative neoplasm-associated myelofibrosis and RBC-transfusion dependence
Source: Leukemia. 2016 Nov 18;31(4):896–902. doi: 10.1038/leu.2016.300 (PMC5383927; doi:10.1038/leu.2016.300)
Supplement: Supplementary Information [file leu2016300x1.doc]

| Supplementary Table 1. Inclusion and exclusion criteria.  Supplementary Table 2. Drug discontinuation guidelines.  Supplementary Table 3. Multivariable analysis of mITT all subjects  Supplementary Table 4. Multivariable analysis of mITT pomalidomide cohort  Supplementary Table 5. Multivariable analysis of mITT placebo cohort  Supplementary Table 6. Frequency of common (≥10%) treatment-related adverse events with a frequency ≥10 percent |
| --- |

**Supplementary Table 1.** Inclusion and exclusion criteria

Inclusion criteria

- - - 1. Age ≥18 y of age at the time of signing the informed consent document.
      2. MPN-associated myelofibrosis (primary myelofibrosis (PMF), post-polycythemia vera myelofibrosis (post-PV MF) and/or post-essential thrombocythemia myelofibrosis (post-ET MF).
      3. RBC-transfusion-dependence:
- Average RBC-transfusion frequency of ≥2 U/28 d over at least the 84 d immediately prior to randomization. There must be no interval >42 d without ≥1 RBC-transfusion.
- Only RBC-transfusions given when the hemoglobin ≤90 g/L[[1]](#footnote-2) are scored in determining eligibility.
- RBC-transfusions due to bleeding are not scored in determining eligibility.
- RBC-transfusions due to chemotherapy-induced anemia are not scored in determining eligibility.
  - - 1. Hemoglobin ≤130 g/L at randomization.
      2. Bone marrow slides that meet defined criteria for central histological review will be submitted to a central reviewer.
      3. A blood cell or bone marrow allo-transplant should not be an appropriate therapy at this time[[2]](#footnote-3).
      4. Erythropoietin should not be an appropriate therapy at this time^2^.
      5. Androgenic steroids should not be an appropriate therapy at this time^2^.
      6. Treatment with systemic corticosteroids is permitted for non-hematological conditions providing the subject is receiving a stable or decreasing dose for ≥84 days immediately prior to randomization and are receiving a constant dose equivalent to ≤10 mg prednisone for the 28 d immediately prior to randomization.
      7. Eastern Cooperative Oncology Group (ECOG) performance score ≤2.
      8. Females of childbearing potential (FCBP) must undergo pregnancy testing and pregnancy results must be negative.
      9. Unless practicing complete abstinence from heterosexual intercourse, sexually active FCPB must agree to use adequate contraceptive methods.
      10. Males (including those who have had a vasectomy) must use barrier contraception (condoms) when engaging in sexual activity with FCBP.
      11. Males must agree not to donate semen or sperm during the duration.
      12. 15. All subjects must:
          - Understand that the investigational product could have a potential teratogenic risk.
          - Agree to abstain from donating blood while taking investigational product and following discontinuation of investigational product.
          - Agree not to share study medication with another person.
          - Be counseled about pregnancy precautions and risks of fetal exposure).
      13. Understand and voluntarily sign an informed consent document before any study related assessments/procedures are conducted.
      14. Able to adhere to the study visit schedule and other protocol requirements.

Exclusion criteria

The presence of any of the following will exclude a subject from enrollment:

1. Prior bone marrow or blood cell transplant.
2. Use of drugs to treat MPN-associated myelofibrosis ≤30 d pre-randomization (42 d for hydroxyurea) or ongoing adverse events from previous treatment.
3. Use of an erythropoietin or androgenic steroids ≤84 d pre- randomization.
4. Anemia from other proved causes other than MPN-associated myelofibrosis.
5. Pregnant or lactating females.
6. More than 10% blasts at any time during the 8 w before randomization unless there is at least one subsequent determination of ≤10% blasts before randomization.
7. Prior history of other cancer unless cancer-free for ≥5 y. Subjects with the following history/concurrent conditions may enroll at any time:

- Basal cell carcinoma of the skin
- Squamous cell carcinoma of the skin
- Prostate cancer stage-1

1. Proved human immunodeficiency virus-1 (HIV-1) infection
2. Active hepatitis-B virus (HBV) or active hepatitis-C Virus (HCV) infection.
3. Prior pomalidomide.
4. Any of the following adverse reactions to prior therapy with thalidomide or lenalidomide
   - Prior ≥grade-2 National Cancer Institute (NCI) Common Terminology Criteria for Adverse Events (CTCAE) allergic reaction to thalidomide and/or lenalidomide
   - Prior desquamating (blistering) rash while taking thalidomide and/or lenalidomide
   - Hypersensitivity to thalidomide and lenalidomide
5. Any of the following laboratory abnormalities:

- Neutrophils <0.5 x 10E+9/L
- Platelets <25 x 10E+9/L
- Estimated glomerular filtration rate <30 mL/min
- Aspartate aminotransferase (AST), and alanine transaminase (ALT) >3.0 x upper limit of normal (ULN)
- Direct bilirubin ≥3 x ULN;
- Uncontrolled hyperthyroidism or hypothyroidism.

1. Deep venous thrombosis (DVT) or pulmonary embolus (PE) <6 mo pre-randomization.
2. Heart disease ≤6 mo pre-randomization including:

- New York Heart Association class >II, congestive heart failure
- Unstable angina
- Myocardial infarction within 6 months

1. Any significant medical condition, laboratory abnormality or psychiatric illness that would prevent the subject from participating in the study.
2. Any condition including the presence of laboratory abnormalities, which places the subject at unacceptable risk if he/she were to participate in the study.
3. Any condition that confounds the ability to interpret data from the study.

Supplementary Table 2. Guidelines for investigational product interruption or discontinuation

| CTCAE v.4 Category | Adverse event | Investigational product |
| --- | --- | --- |
| Immune system disorders | Allergic reaction/hypersensitivity (including drug fever) ≥grade-2 | Discontinue |
| Blood and lymphatic system disorders | Granulocytes <0.5x10E+9/L  (grade-4) | Interrupt. Restart once granulocytes ≥0.5 x 10E+9/L |
| Blood and lymphatic system disorders | Platelets <25x10E+9/L | Interrupt. Restart once platelets ≥25 x 10E+9/L |
| Vascular disorders | Thrombosis/embolism  Grade-3 or -4 | Discontinue |
| Skin and subcutaneous tissue disorders | Desquamating/blistering rash (Stevens-Johnson, toxic epidermal necrolysis) | Discontinue |
| Skin and subcutaneous tissue disorders | Rash: erythema multiforme | Discontinue |
| Nervous system disorders | Neuropathy – cranial/motor/sensory  Grade-2 | Interrupt. Restart once adverse event is completely resolved. |
| Nervous system disorders | Neuropathy – Cranial/motor/sensory  ≥Grade 3 | Discontinue |

**Supplementary Table 3**. Logistic regression analysis^1^ of response for all subjects (modified *intent-to-treat* population)

|  | **Univariable model** | | | **Final model** | | |
| --- | --- | --- | --- | --- | --- | --- |
|  | **Odds ratio** | **95% CI** | **P-value** | **Odds ratio** | **95% CI** | **P-value** |
| **Age (y)**  **(≤65 *vs*. >65)** | 1.40 | 0.68, 2.87 | 0.37 |  |  |  |
| **Sex (M/F)** | 0.98 | 0.44, 2.16 | 0.95 |  |  |  |
| **WBC (x10E+9/L)**  **(<25 *vs.* ≥25)** | 0.74 | 0.28, 1.95 | 0.54 |  |  |  |
| **RBC transfusions (/28 d)^2^**  **(low *vs.* high)** | 3.94 | 1.34, 11.60 | 0.01 | 3.94 | 1.34, 11.60 | 0.01 |
| **Spleen size (cm)**  **(<12 *vs.* ≥12)** | 1.36 | 0.65, 2.87 | 0.41 |  |  |  |
| **Subjects per site**  **(<5 *vs.* ≥5)** | 0.67 | 0.33, 1.37 | 0.27 |  |  |  |
| **Interval diagnosis to randomization (y)**  **(<2 *vs.* ≥2)** | 0.72 | 0.36, 1.46 | 0.36 |  |  |  |
| **Primary myelofibrosis**  **(Y/N)** | 1.87 | 0.74, 4.74 | 0.190 |  |  |  |
| **Therapy**  **(pomalidomide *vs.* placebo)** | 1.07 | 0.50, 2.26 | 0.87 |  |  |  |

Abbreviations: CI, confidence interval.

^1^A logistic regression model is used to identify the prognostic factors for the clinical response. Final model only includes the most significant prognostic factors after using backward elimination method to remove less significant prognostic factors.

^2^For EU, America and Australia, Low<4 units RBC/28 d; High ≥4 units RBC/28 d; for China, Low <5 units RBC/28 d; High ≥5 units RBC/28 d, for Japan Low <8 units RBC/28 d; High ≥8 units RBC/28 d.

**Supplementary Table 4.** Logistic regression analysis^1^ of response for subjects randomized to receive pomalidomide (modified *intent-to-treat* population)

|  | **Univariable model** | | | **Final model** | | |
| --- | --- | --- | --- | --- | --- | --- |
|  | **Odds ratio** | **95% CI** | **P-value** | **Odds ratio** | **95% CI** | **P-value** |
| **Age (y)**  **(≤65 *vs.* >65)** | 2.36 | 0.99, 5.62 | 0.05 | 2.260 | 0.93, 5.50 | 0.07 |
| **Sex (Male/Female)** | 0.60 | 0.24, 1.54 | 0.29 |  |  |  |
| **WBC (x10E+9/L)**  **(<25 *vs.* ≥)** | 1.13 | 0.31, 4.20 | 0.85 |  |  |  |
| **RBC transfusions (/28 d)**  **(low *vs.* high)^2^** | 3.13 | 0.88, 11.09 | 0.077 | 3.07 | 0.85, 11.06 | 0.09 |
| **Spleen size (cm)**  **(<12 *vs.* ≥12)** | 1.54 | 0.61, 3.90 | 0.36 |  |  |  |
| **Subjects per site**  **(<5 *vs.* ≥5)** | 0.88 | 0.37, 2.08 | 0.77 |  |  |  |
| **Interval diagnosis to randomization (y)**  **(<2 *vs.* ≥2)** | 1.09 | 0.45, 2.61 | 0.85 |  |  |  |
| **Primary myelofibrosis**  **(Y/N)** | 2.90 | 0.82, 10.29 | 0.103 | 2.62 | 0.73, 9.49 | 0.14 |

Abbreviations: CI, confidence interval.

^1^A logistic regression model is used to identify the prognostic factors for the clinical response. Final model only includes the most significant prognostic factors after using backward elimination method to remove less significant prognostic factors.

^2^For EU, America and Australia, Low<4 units RBC/28 d; High ≥4 units RBC/28 d; for China, Low <5 units RBC/28 d; High ≥5 units RBC/28 d, for Japan Low <8 units RBC/28 d; High ≥8 units RBC/28 d.

**Supplementary Table 5. Logistic regression analysis^1^ of response for subjects randomized to receive placebo (modified intent-to-treat population)**

|  | **Univariable model** | | | **Final model** | | |
| --- | --- | --- | --- | --- | --- | --- |
|  | **Odds ratio** | **95% CI** | **P-value** | **Odds ratio** | **95% CI** | **P-value** |
| **Age (y)**  **(≤65 *vs.* >65)** | 0.38 | 0.07, 1.81 | 0.22 |  |  |  |
| **Sex (M/F)** | 2.74 | 0.55, 13.58 | 0.22 |  |  |  |
| **WBC (10E+9/L)**  **(<25 *vs.* ≥25)** | 0.36 | 0.08, 1.66 | 0.19 | 0.21 | 0.04, 1.22 | 0.08 |
| **RBC transfusions (/28 d)**  **(low *vs.* high)^2^** | 6.44 | 0.78, 53.01 | 0.08 | 8.61 | 0.90, 82.34 | 0.06 |
| **Spleen size (cm)**  **(< 12 *vs.* ≥12)** | 1.09 | 0.31, 3.80 | 0.89 |  |  |  |
| **Subjects per site**  **(<5 *vs.* ≥5)** | 0.38 | 0.10, 1.38 | 0.14 |  |  |  |
| **Interval diagnosis to randomization (y)**  **(<2 *vs.* ≥2)** | 0.30 | 0.08, 1.10 | 0.07 | 0.21 | 0.05, 0.92 | 0.05 |
| **Primary myelofibrosis**  **(Y/N)** | 0.90 | 0.22, 3.80 | 0.89 |  |  |  |

Abbreviations: CI, confidence interval.

^1^A logistic regression model is used to identify the prognostic factors for the clinical response. Final model only includes the most significant prognostic factors after using backward elimination method to remove less significant prognostic factors.

^2^For EU, America and Australia, Low<4 units RBC/28 d; High ≥4 units RBC/28 d; for China, Low <5 units RBC/28 d; High ≥5 units RBC/28 d, for Japan Low <8 units RBC/28 d; High ≥8 units RBC/28 d.

**Supplementary Table 6.** Frequency of common (≥10%) treatment-emergent adverse events and thrombo-embolic events.

|  | **Pomalidomide (%)** | **Placebo (%)** | **P-value** |
| --- | --- | --- | --- |
| Edema (peripheral)  Fatigue  Pyrexia  Asthenia  Diarrhea  Dyspnea  Neutropenia  Constipation  Cough  Nausea  Thrombocytopenia  Vomiting  Decreased appetite  Dizziness  Thrombo-embolism | 32  20  19  13  19  16  16  14  14  11  13  10  11  10  12 | 17  19  15  10  22  11  6  11  11  19  15  12  8  15  7 | 0.02  1.00  0.38  0.54  0.74  0.34  0.04  0.55  0.55  0.08  0.85  0.67  0.66  0.40  0.27 |

1. For countries following Nordic guidelines, haemoglobin levels for RBC-transfusions should not exceed 100 g/L haemoglobin. [↑](#footnote-ref-2)
2. Appropriate means the anticipated benefits of the proposed therapy exceed the anticipated risks by a sufficient margin that the proposed therapy should be given. [↑](#footnote-ref-3)
